# PPARγ stimulates expression of L-type amino acid and taurine transporters in human placentas: the evidence of PPARγ regulating fetal growth

**DOI:** 10.1038/srep12650

**Published:** 2015-07-31

**Authors:** Zhaoguang Chen, Ping He, Xiaoying Ding, Ying Huang, Hang Gu, Xin Ni

**Affiliations:** 1 Department of Physiology, Second Military Medical University, Shanghai, China; 2 Maternity and Child Health Hospital of Pudong New District, Shanghai, China; 3 Department of Obstetrics and Gynecology, Changhai Hospital, Shanghai, China

## Abstract

Placental amino acid transporters and peroxisome proliferator-activated receptors (PPARs) have been implicated to placental development and therefore regulation of fetal growth. We analyzed the correlation between the expression of amino acid transporters and PPARs and investigated whether PPARs control the expression of amino acid transporters in placentas. It was found that protein expression of PPARγ and L-type amino acid transporter 1(LAT1) and 2 (LAT2) was decreased in small-for-gestational-age (SGA) placentas. LAT1, LAT2 and taurine transporter (TAUT) expression correlated to PPARγ level and birth weight. In cultured placental cells, PPARγ agonist stimulated LAT1 and LAT2 and TAUT, which was reversed by PPARγ siRNA. PPARγ up-regulation of LAT1 and TAUT was through specificity protein 1 (Sp-1) while stimulation of LAT2 expression was via induction of gene transcription. Our data suggest that PPARγ, SP-1, LAT1 and LAT2 in placentas are involved in control of fetal growth. PPARγ signaling pathway may be the therapeutic target for intrauterine growth restriction.

The exchange of nutrients across the placenta is indispensable for fetal growth over the course of gestation. The dysfunction of placental transportation is considered to be one of major cause of intrauterine growth restriction (IUGR), a pregnancy complication which is associated with perinatal morbidity and mortality^1^ and now regarded as a risk factor for diabetes and cardiovascular disease in adult age^2^.

Amino acids are essential for synthesis of protein and therefore play an important role in fetal growth. In addition, Amino acids have also been implicated to be involved in the process of implantation and placentation^3^. The smooth transportation of amino acids via the placenta relies on cooperation of various transporters in the syncytiotrophoblasts^4^,^5^. A number of amino acid transporters have been identified in placenta. Among them, system A, L and β amino acid transporters have been implicated to be involved in fetal growth and pathogenesis of IUGR^6^,^7^,^8^,^9^. System A amino acid transporters which transport neutral amino acids with short side chains consist of three isoforms SNAT1 (SLC38A1), SNAT2 (SLC38A2) and SNAT4 (SLC38A4)^10^. System L which has two major isoforms, LAT1 (SLC7A5) and LAT2 (SLC7A8) is Na^+^ -independent transport system and mediates transport of branched-chain and aromatic amino acids^11^. System β transporters transport β-amino acids including taurine. Since taurine is an important nutrient in intrauterine life and required for fetal organ development and cellular renewal of syncytiotrophoblast^12^,^13^, the taurine transporter (TAUT) is the most studied one in human placentas. The activity of system A, L and TAUT is significantly downregulated in IUGR placentas^6^,^7^,^8^,^9^, and system A activity correlates to the severity of IUGR^6^. So far, there are few studies regarding the expression and the regulatory mechanisms of these transporters in human placenta.

Peroxisome proliferator activated receptors (PPARs), the ligand-inducible transcription factors, belong to the nuclear receptor superfamily. They have been implicated to play important roles in cell differentiation and proliferation, lipid metabolism, inflammation and maintenance of physiological energy homeostasis^14^,^15^. In mammals, three major PPAR isoforms have been identified, PPARα, PPARβ/δ and PPARγ, with different tissue distribution and function^14^,^15^. In human placenta, three PPARs have been found, however, the physiological roles of them have not been fully understood. PPARγ and PPARβ appear to have a pivotal role in placental development^16^. PPARγ has been implicated to regulate trophoblast differentiation and maturation^17^ as well as play a predominant role in regulation of fat accumulation in trophoblasts and transport of fatty acids from the placenta to the fetus^18^. Recently, several studies have implicated that the expression level of PPARs might be associated with IUGR. For instance, Díaz *et al.*^19^ demonstrated that PPAR γ mRNA level is reduced in placentas of small-for-gestational age (SGA) fetuses and correlated to fetal and placental weights.

Given that the essential function of PPARs is regulation of gene transcription, we hypothesized that PPARs might modulate the expression of amino acid transporters in placentas, thereby controlling fetal growth. To test these, we determined the expression of three PPARs and system A, L and TAUT in human placentas of SGA, large- (LGA) and appropriate-for-gestational age (AGA) fetuses and analyzed the correlation between the expression of PPARs and level of system A, L and TAUT or the birth weight. Then, using cultured placental cells as a model, we investigated the effects of PPARs on the expression of above amino acid transporters and subsequently elucidated the molecular mechanisms underlying PPARs modulation of amino acid transporters.

## Results

### The expression of LAT1 and LAT2 is significantly decreased in SGA placentas and correlates to PPARγ level and birth weight

**Figure 1:**
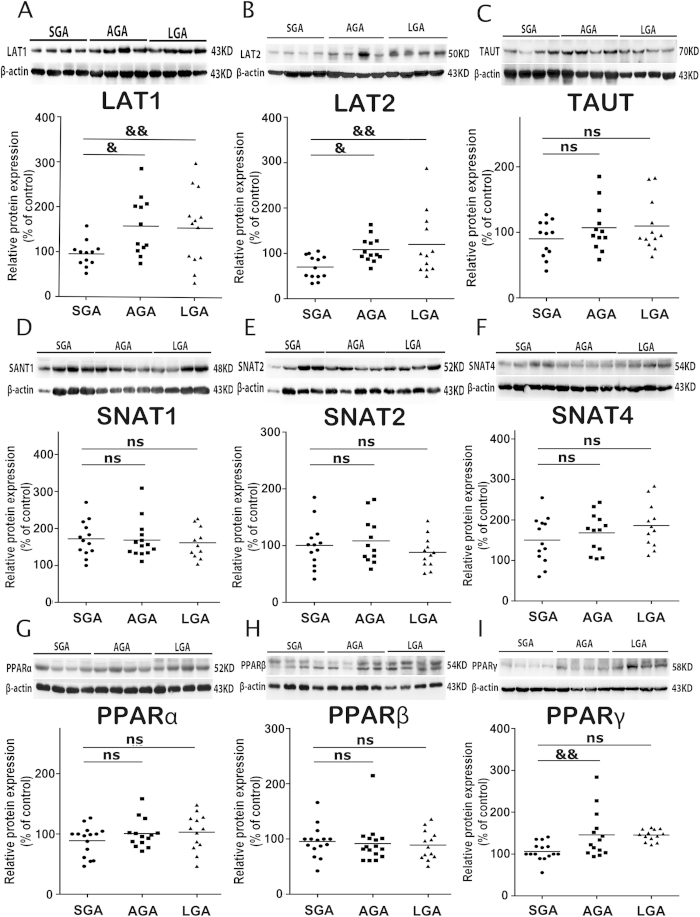
The expression of amino acid transports including system A, L and TAUT and the expression of PPARs in SGA, AGA and LGA placentas. Placental tissues were collected at term delivery of singleton infants who were born SGA (n = 16), AGA (n = 17) and LGA (n = 14). The protein levels of LAT1 (**A**), LAT2 (**B**), TAUT (**C**), SNAT1 (**D**), SNAT2 (**E**), SNAT4 (**F**), PPARα (**G**), PPARβ (**H**) and PPARγ (**I**) were determined by western blotting. Data were presented as mean ± SEM. Representative protein bands were presented on the top of the histogram. &*P* < 0.05; &&*P* < 0.01. ns: no significance.

As shown in Fig. 1, the protein expression of LAT1 and LAT2 in the placentas was significantly lower in SGA group than in AGA and LGA groups (*P* < 0.01), whereas the levels of system A transporters (SNAT1, SNAT2 and SNAT4) and TAUT were not differed among SGA, AGA and LGA groups.

The protein level of PPARγ was significantly down-regulated in SGA group compared with AGA and LGA groups (*P* < 0.01). There was no significant difference in PPAR α and PPAR β/δ expression among SGA, AGA and LGA groups.

Correlation analysis showed that LAT1, LAT2 and PPARγ levels correlated to birth weight (*P* < 0.01, supplement Fig. 1). PPARγ expression correlated to LAT1, LAT2 and TAUT level (*P* < 0.01, supplement Fig. 1), indicating that LAT1, LAT2 and TAUT might be regulated by PPAR γ.

### PPARγ increases LAT1, LAT2 and TAUT expression in cultured placental trophoblasts

**Figure 2:**
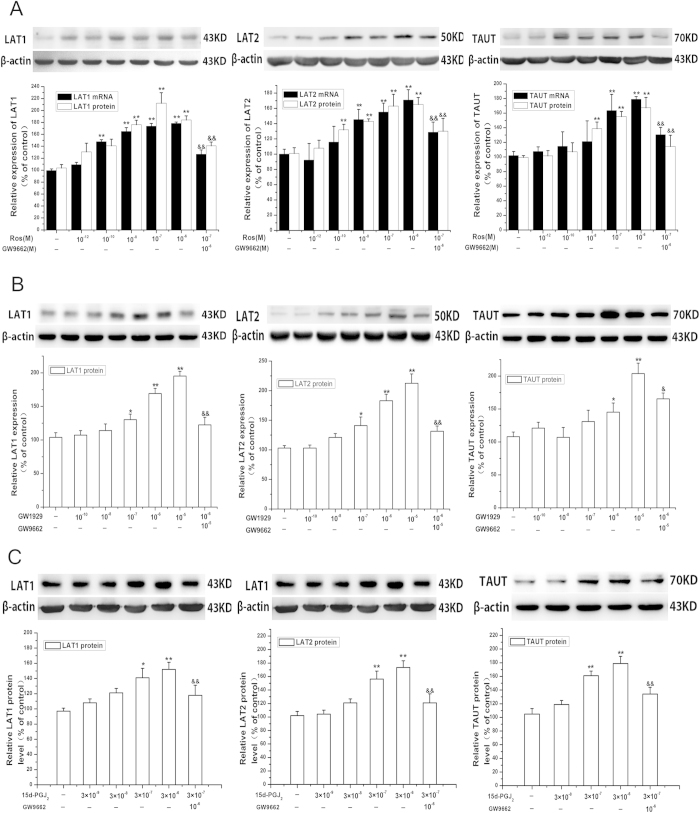
PPARγ stimulates the expression of LAT1, LAT2 and TAUT in cultured placental trophoblast cells. (**A**) the effects of rosiglitazone on the expression of LAT1, LAT2 and TAUT. Primary trophoblast cells were treated with increasing concentration of rosiglitazone (10^−12^–10^−6^ M) for 24 h in the presence or absence of PPARγ antagonist GW9662 (10^−6^ M) for 24 h. The cells were then harvested for determination of LAT1, LAT2 and TAUT mRNA and protein levels as described in *Methods.* Representative protein bands are presented on the top of corresponding histogram. Data are presented as mean percent control ± SEM of five cultures (n = 5) performed in triplicate. Ros, rosiglitazone. *P < 0.05, **P < 0.01 vs control; &&*P* < 0.01 vs Ros. B &C, Ros. B &C, the effects of GW1929 (**B**) and 15d-PGJ2 (**C**) on LAT1, LAT2 and TAUT expression. Trophoblast cells were treated with increasing concentration of GW1929 (10^−9^–10^−5^ M) or 15d-PGJ2 (3 × 10^−9^–3 × 10^−6^ M) for 24 h in the presence or absence of GW9662 (10^−6^ M) for 24 h. The cells were then harvested for determination of LAT1, LAT2 and TAUT mRNA and protein levels. Data are presented as mean percent control ± SEM of four cultures (n = 4) performed in triplicate. Representative protein bands are presented on the top of corresponding histogram.*P < 0.05, **P < 0.01 vs control; &*P* < 0.05; &&*P* < 0.01 vs GW1929 or 15d-PGJ2.

Treatment of the cells with increasing concentration of PPARγ agonist rosiglitazone (10^−12^–10^−6^M) for 24 h resulted an increase in LAT1, LAT2, TAUT mRNA and protein expression in a dose-dependent manner (Fig. 2A). Time-dependent experiment showed that the maximal expression of LAT1, LAT2 and TAUT was occurred at 24 h after rosiglitazone (10^−7^ M) treatment (supplement Fig. 2). The effect of rosiglitazone on LAT1, LAT2 and TAUT could be blocked by PPARγ antagonist GW9662 (10^−6^ M) (Fig. 2A). Rosiglitazone treatment did not affect mRNA and protein expression of SNAT1, SNAT2 and SNAT4 (supplement Fig. 3).

In order to verify the effects of PPARγ on the transporters, the nature-ligand of PPARγ 15-Deoxy-∆^12^,^14^-Prostaglandin J_2_ (15d-PGJ_2_) and another synthetic high-selective agonist of PPARγ GW1929, were applied. As shown in Fig. 2B,C, both GW1929 and 15d-PGJ_2_ up-regulated protein expression of LAT1, LAT2 and TAUT. GW9662 reversed the effects of 15d-PGJ_2_ and GW1929.

**Figure 3:**
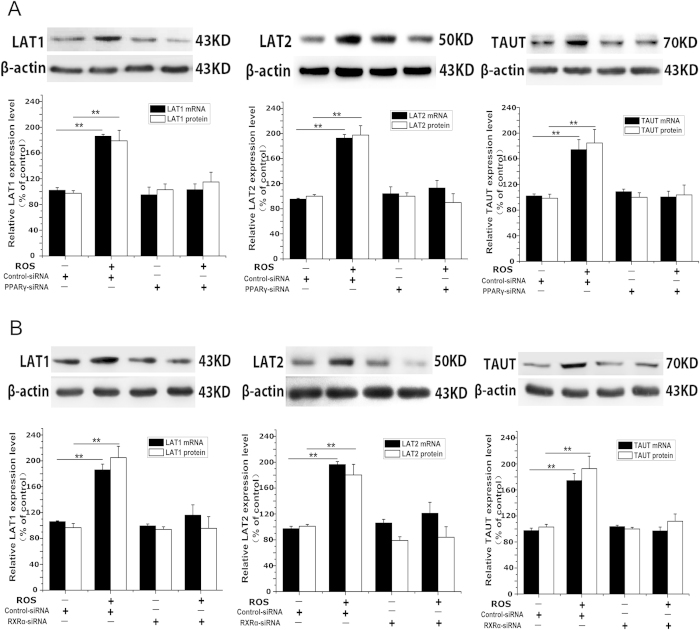
The effects of PPARγ siRNA and RXRα siRNA on the expression of LAT1, LAT2 and TAUT induced by PPARγ agonist. (**A**) the effects of rosiglitazone on LAT1, LAT2 and TAUT expression in PPARγ knockdown cells. Primary trophoblast cells were transfected with PPARγ siRNA or scramble siRNA and then treated with rosiglitazone (10^−6^ M) for 24 h as described in Methods. The cells were then harvested for determination of LAT1, LAT2 and TAUT mRNA and protein levels and the protein level of PPARγ. Representative protein bands of LAT1, LAT2 and TAUT are presented on the top of corresponding histogram. Data are presented as mean percent control ± SEM of four cultures (n = 4) performed in triplicate. Ros, rosiglitazone., **P < 0.01 vs vehicle. (**B**) the role of RXRα in PPARγ modulation of LAT1, LAT2 and TAUT expression. Trophoblast cells were transfected with RXRα siRNA or scramble siRNA and then treated with rosiglitazone (10^−6^ M) for 24 h. LAT1, LAT2 and TAUT mRNA and protein levels in cells were determined by real-time RT-PCR and western blot analysis, respectively. The protein level of RXRα was determined by western blotting. Representative protein bands of LAT1, LAT2 and TAUT are presented on the top of corresponding histogram. Data are presented as mean percent control ± SEM of four cultures (n = 4) performed in triplicate. Ros, rosiglitazone. **P < 0.01 vs vehicle.

Then siRNA approach was applied to verify the above results. Transfection of the cells with siRNA targeting PPARγ resulted in about 85% reduction of PPARγ expression (supplement Fig. 4). PPARγ siRNA reversed the effects of rosiglitazone on the expression of LAT1, LAT2 and TAUT (Fig. 3A).

### PPARγ stimulation of LAT1, LAT2 and TAUT expression requires RXRα

Classically, PPARγ regulates target gene transcription by forming heterodimer with RXR^20^. RXRα is the predominant isoform of RXR in human placenta^21^. Then we investigated whether RXRα is involved in PPARγ modulation of LAT1, LAT2 and TAUT expression. Transfection of RXRα siRNA caused 90% reduction in RXRα expression (supplement Fig. 4). Rosiglitazone-induced LAT1, LAT2 and TAUT expression was not occurred in cells transfected with RXRα siRNA (Fig. 3B).

### PPARγ modulation of LAT1 and TAUT expression requires *de novo* protein synthesis while it stimulates LAT2 expression via increasing gene transcription

**Figure 4:**
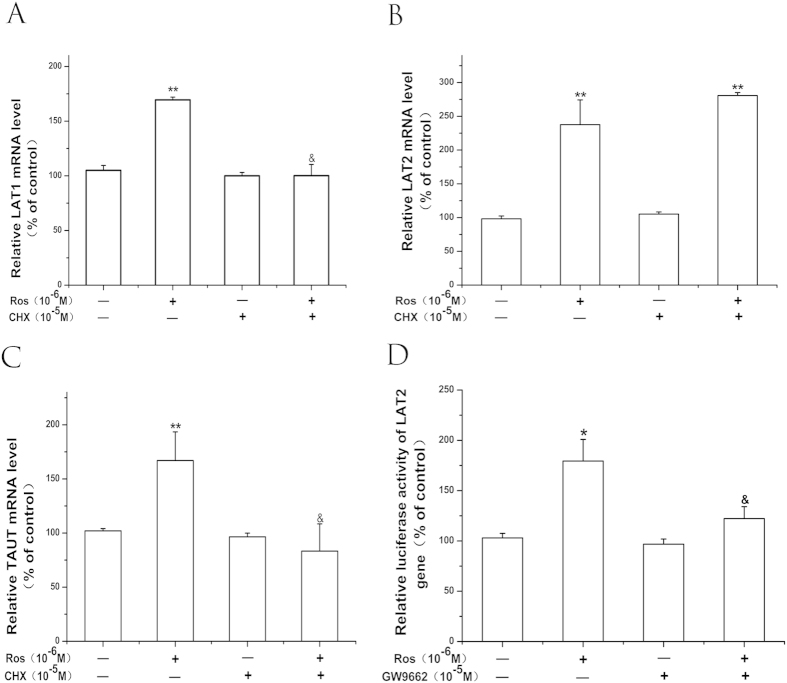
PPARγ modulation of LAT1 and TAUT expression requires *de novo* protein synthesis while it directly stimulates LAT2 gene transcription. (**A–C**) the effect of protein inhibitor CHX on PPARγ-induced alternation of LAT1 (**A**) LAT2 (**B**) and TAUT (**C**) mRNA. Trophoblasts were pretreated with CHX (10^−5^ M) for 1 h, then treated with rosiglitazone (10^−6^ M) for 24 h. The level of LAT1, LAT2 and TAUT mRNA was assessed by real-time RT-PCR. Data are presented as mean percent control ± SEM of four cultures (n = 4) performed in triplicate. Ros, rosiglitazone. **P < 0.01 vs vehicle. &*P* < 0.05 vs Ros. (**D**) the effect of rosiglitazone on LAT2 promoter activity. Placental cells were transfected with LAT2 reporter plasmid and then treated with rosiglitazone (10^−6^ M) in the absence or presence of GW9662 (10^−6^ M) for 24 h. Values represent the mean ± SEM (n = 4). Ros, rosiglitazone. *P < 0.05 vs vehicle; &P < 0.05 vs Ros.

We investigated whether this process requires *de novo* protein synthesis using protein synthesis inhibitor CHX. Trophoblast cells were pretreated with CHX (10^−5^ M) for 1 h, then incubated with PPARγ agonists rosiglitazone (10^−6^ M) for 24 h. CHX abolished stimulatory effect of rosiglitazone on LAT1 and TAUT expression but did not affect rosiglitazone–induced LAT2 expression (Fig. 4A–C).

We then investigated whether PPARγ-induced up-regulation of LAT2 is associated with stimulation of LAT2 gene transcription. As shown in Fig. 4D, Rosiglitazone (10^−6^ M) significantly increased LAT2 transcription. This effect was blocked by the PPARγ antagonist GW9662.

### PPARγ stimulation of LAT1 and TAUT expression is dependent on SP-1 signaling

As shown above, PPARγ stimulation of LAT1 and TAUT expression requires *de novo* protein synthesis. We then explored which protein is involved in this process. The results from bioinformatics website JASPAR (http://jaspar.genereg.net/) indicated that a number of SP-1 binding sites are located in the promoter of LAT1 and TAUT genes (LAT1: −1600 ~ −20 bp; TAUT: −2000 ~ −1800 bp, −1200 ~ −700 bp, −100 ~ 0 bp). Our previous study has demonstrated that PPARγ stimulates SP-1 expression and SP-1 mediates PPARγ upregulation of 11-βHSD-2 expression in human placenta^22^.

**Figure 5:**
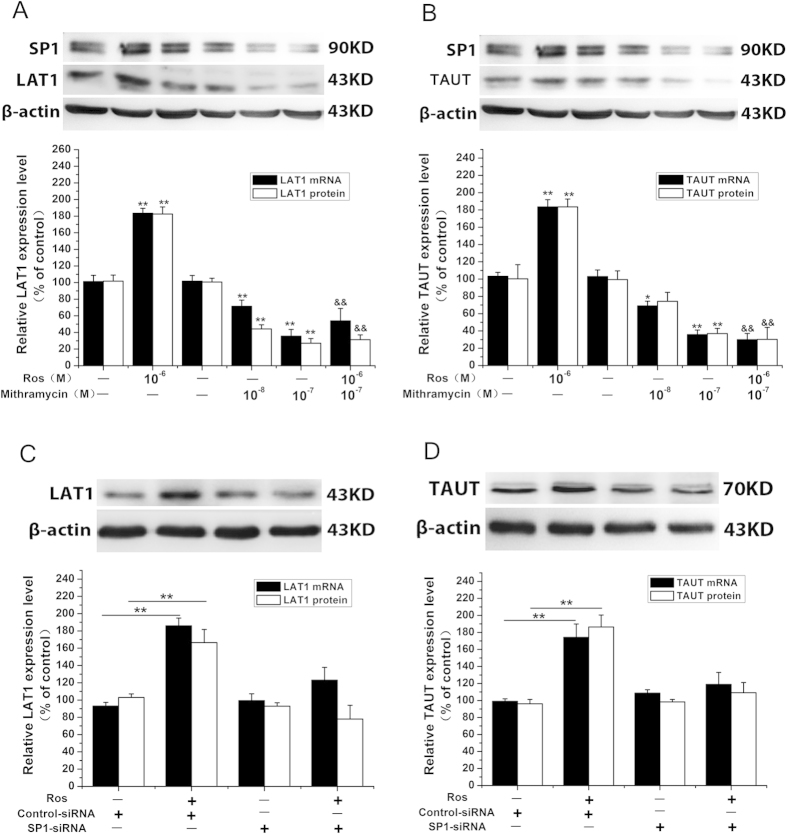
PPARγ modulation of LAT1 and TAUT expression requires SP-1 in placental cells. (**A**) the effect of SP-1 inhibitor on PPARγ- induced alternation of LAT1 and TAUT expression. Trophoblast cells were treated with rosiglitazone (10^−6^ M) in the presence or absence of SP-1 inhibitor mithramycin A (10^−7^ M) for 24 h. The cells were then harvested for determination of LAT1 and TAUT mRNA and protein expression. Representative protein bands of LAT1 and TAUT are presented on the top of corresponding histogram. Data are presented as mean percent control ± SEM of four cultures (n = 4) performed in triplicate. Ros, rosiglitazone. *P < 0.05, **P < 0.01 vs vehicle. &&*P* < 0.01 vs Ros. (**B**) the effects of PPARγ on LAT1 and TAUT expression in SP-1 knockdown cells. Cells were transfected with SP-1 siRNA or scramble siRNA and then treated with rosiglitazone (10^−6^ M) for 24 h. The cells were then harvested for determination of LAT1 and TAUT mRNA and protein expression as well as the protein expression of SP-1. Representative protein bands of LAT1 and TAUT are presented on the top of corresponding histogram. Data are presented as mean percent control ± SEM of four cultures (n = 4) performed in triplicate. Ros, rosiglitazone. **P < 0.01.

We therefore investigated whether SP-1 is involved in PPARγ modulation of LAT1 and TAUT expression. As shown in Fig. 5A,B, application of SP-1 inhibitor mithramycin A (10^−8^–10^−7^ M) also reversed the effect of PPARγ on LAT1 and TAUT. In consistent with the above results, SP-1 siRNA abolished the stimulatory actions of PPAR γ on LAT1 and TAUT expression (Fig. 5C,D). We also confirmed that SP-1 siRNA caused a 75% decrease in SP-1 expression (supplement Fig. 4).

**Figure 6:**
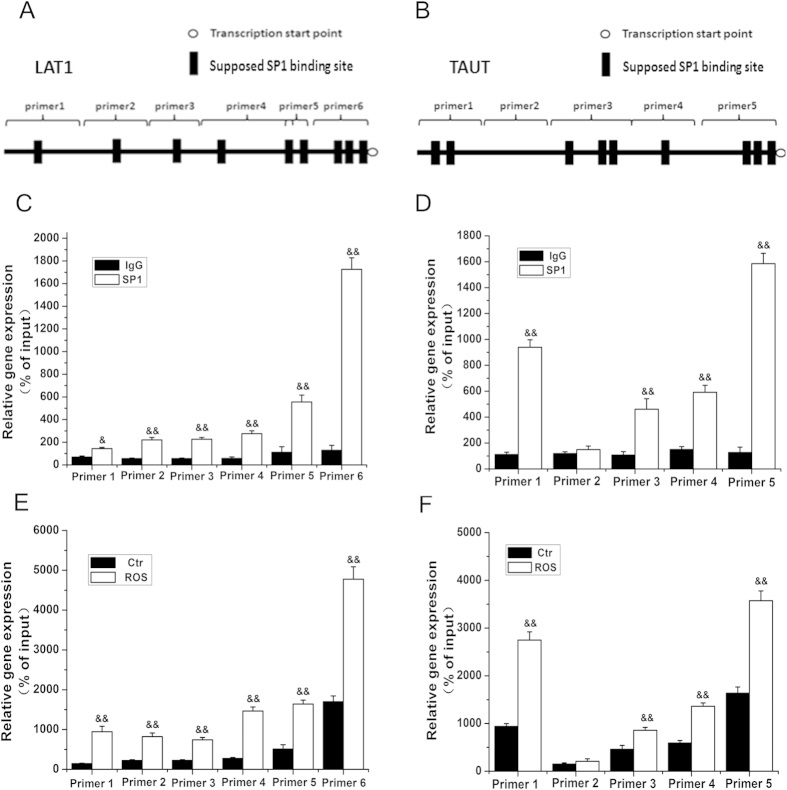
PPARγ modulates LAT1 and TAUT expression by increasing SP-1 binding activity in the LAT1 and TAUT gene promoter. Cells were treated with rosiglitazone (10^−6^ M) or with vehicle for 24 h. ChIP was then applied to determine SP-1 binding activity in LAT1 and TAUT promoter. (**A,B)** schematic demonstration of the primers used for LAT1 and TAUT promoter. (**C**,**D**) the basal SP-1 binding activity of LAT1(**C**) and TAUT (**D**) promoter. (**E,F**) the SP-1 binding activity of LAT1(**E**) and TAUT (**F**) promoter in response to rosiglitazone (10^−6^ M) treatment. Data are presented as mean percent control ± SEM of four cultures (n = 4) performed in triplicate. Ros, rosiglitazone. &&*P* < 0.01 vs IgG or control.

In order to verify that PPAR increases LAT1 and TAUT expression through enhancement of SP-1 binding to the promoter of LAT1 and TAUT, ChIP assays were carried out. We designed 6- and 5-pairs primers to cover the promoter of LAT1 and TAUT gene, respectively (Fig. 6A,B). The results showed that, in physiological conditions, SP-1 was recruited to the LAT1 and TAUT promoter in trophoblast cells. Rosiglitazone treatment enhanced SP-1 binding to the promoter of LAT1 and TAUT (Fig. 6C–F).

### The SP-1 expression is downregulated in SGA placentas

**Figure 7:**
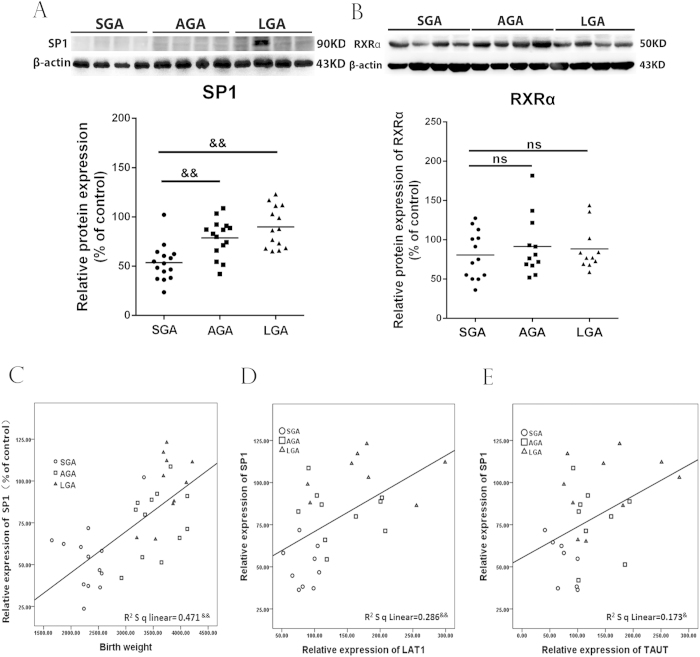
The expression of SP-1 and RXRα in SGA, AGA and LGA placentas and correlation of SP-1 level with LAT1, TAUT and birth weight. (**A,B**) the expression of SP-1(**A**) and RXRα (**B**) in placentas. Placental tissues were collected at term delivery of singleton infants who were born SGA (n = 16), AGA (n = 17) and LGA (n = 14). The protein levels of SP-1 and RXRα were determined by western blotting. Representative protein bands were presented on the top of histogram. Data were presented as mean ± SEM. (**C–E**) the correlation between SP-1 with birth weight as well as LAT1 and TAUT expression. &*P* < 0.05; &&*P* < 0.01, ns: no significance.

Since RXRα and SP-1 are involved in PPARγ regulation of amino acid transporters, we examined RXRα and SP-1 expression in placentas of SGA, LGA and AGA. As shown in Fig. 7, the expression of SP-1 was significantly down-regulated in placentas of SGA infants compared with placentas from AGA and LGA infants (*P* < 0.01), whereas the level of RXRα was not differed among SGA, LGA and AGA groups. Correlation analysis showed that SP-1 positively correlated to birth weight and protein expression of LAT1 and TAUT.

## Discussion

The present study demonstrated that PPARγ modulates the expression of amino acid transporters in human placenta and decreased expression of LAT1 and LAT2 in SGA is associated with down-regulation of PPARγ expression.

A number of studies have shown that PPARs might be involved in various pregnancy complications, such as preeclampsia and gestational diabetes mellitus (GDM). McCarthy *et al.*^23^,^24^ have demonstrated that antagonism of PPARγ in rats during pregnancy can induce features of preeclampsia characterized by maternal hypertension, proteinuria and endothelial dysfunction, while the PPARγ agonist, rosiglitazone, can ameliorate the features of preeclampsia in the animal model of reduced uterine perfusion pressure. Moreover, PPARγ expression is significantly reduced in preeclamptic placentas^22^. Some studies reported that PPARγ protein expression is downregulated in placentas of pregnant women with GDM and an animal model of GDM^25^. For IUGR, there might be controversy about the expression of PPARs in placentas. Holdsworth-Carson *et al.*^26^ reported that mRNA and protein level of PPARγ is not differed between IUGR and normal placentas. Recently, Díaz and coworkers^19^ have demonstrated that PPARγ mRNA expression is lower in SGA placentas based on a relative large population (116 patients), and proposed that PPARγ expression in placenta is associated with fetal growth. In consistent with the study of Díaz *et al.*, we showed that PPARγ protein expression was significantly lower in SGA placentas than that in AGA placentas and correlated to the birth weight. Moreover, we demonstrated that PPARγ expression correlated to amino acid transporters LAT1, LAT2 and TAUT, and in cultured placental cells, we confirmed that PPARγ stimulated LAT1, LAT2 and TAUT expression Taken together, we suggest that PPARγ is an important factor that increases the expression of amino acid transporters, thereby being involved in control of fetal growth during pregnancy.

There are a few studies about the expression of amino acid transporters in human placentas. Malina *et al.*^27^ reported that no difference in SNAT1 and SNAT2 mRNA expression was found between the IUGR group and normal group. The study of Roos and coworkers showed that TAUT protein expression did not differ among IUGR placentas and control ones although the activity of TAUT was lower in IUGR placentas^28^. In the present study, we also found that the expression of TAUT was not significantly different among SGA, LGA and AGA groups. As mentioned, we actually found that LAT1 and LAT2 protein expression was markedly decreased in placentas from SGA fetuses, which might be consistent with the studies of Jansson’s group where they have shown that activity of system L is also down-regulated in IUGR placentas^8^,^9^. More recently, Aiko *et al.*^29^ have shown that the expression level of LAT1 in microvillous syncytiotrophoblasts is increased in IUGR placentas as compared to term placentas by using immunocytochemistry. It should be pointed out that amino acid transporter activity of placental tissues might not be positively correlated to expression of transporters due to trafficking of transporters between intracellular stores and the plasma membrane where they function. A number of studies have revealed that insulin could stimulate translocation of SNAT2 to cell membrane in adipocytes and muscle cells, which accounts for insulin stimulation of amino acid transportation^30^, and mTOR modulates amino acids uptake via regulating cell surface abundance of system A and L transporters in trophoblast cells^31^. Thus, it is clear that both the global expression and translocation of amino acid transporters can influence their activity. Thus, further research is required to explore the translocation and localization of these transporters in SGA placentas.

Rosiglitazone belongs to thiazolidinediones (TZDs), the synthetic compounds which are applied to treat type II diabetes^32^. In recent year, a number of studies demonstrated that many effects of TZDs are not PPARγ-dependent. The evidence from Palakurthi *et al.*’s study^33^ shown that the TZDs inhibition of proliferation and tumor growth of embryonic stem cells is PPARγ-independent. Gras *et al.*^34^ have demonstrated that TZDs induce proliferation of human bronchial epithelial cells through the GPR40 receptor. Thus, other PPARγ agonists as well as siRNA targeting PPARγ were applied to elucidate whether the up-regulatory effect of rosiglitazone on LAT1, LAT2 and TAUT is PPARγ-dependent, and the results showed that the effects of rosiglitazone is PPARγ-dependent.

As mentioned, PPARs control the transcription of target genes by binding to specific PPRE motifs. However, many researches revealed the mechanism underlying modulation of gene transcription by PPARγ is far more complicated than our previous views. Some studies have shown that PPARs can transrepress the expression of non-PPRE containing genes^21^,^35^, indicating that PPRE is not indispensable element in PPAR modulation of target gene transcription. In the present study, we found that PPARγ modulation of LAT1, LAT2 and TAUT were through different mechanisms. PPARγ may directly stimulate LAT2 transcription, whereas it regulates LAT1 and TAUT gene expression via SP-1, a nuclear transcription factor. Bioinformatics reveals the presence of putative PPRE-like motifs in LAT2 gene promoter region. Nevertheless, additional studies are required to confirm whether PPARγ stimulates LAT2 expression via binding to PPRE-like sequence in its promoter. SP-1, an important transcription factor in human placentas, plays a pivotal role in invasion and differentiation of trophoblast cells^36^. More recently, SP-1 has also been shown to be participated in modulation of 11β-HSD2 in human syncytiotrophoblasts^22^,^37^. In the present study, we showed that SP-1 protein expression is decreased in SGA placentas and positively correlates to LAT1 and TAUT as well as birth weights. These data suggest that SP-1 might play a critical role in fetal growth via regulation of amino acid transportation cross placenta.

The present study appears to be the first that assessed the correlation of expression of three PPARs and amino acid transporters in term, singleton and uncomplicated pregnancies and determined PPARγ regulation of the transporters in placentas. One of the limitations is that we did not assess the activity and expression of amino acid transporters expression and did not perform PPARγ DNA binding studies in placental tissues which might provide direct evidence for PPARγ regulation of amino acid transporters.

In the present study, it was found that the maternal weight and BMI were significantly lower in the patients with SGA as compared to those with AGA. Thus, the occurrence of SGA in the patients recruited in this study could also be associated with maternal nutrient state. A number of studies have demonstrated that maternal nutrient restriction would lead to reduced expression of nutrient transporters including amino acid transporters^38^.

In conclusion, the present study demonstrated that PPARγ stimulates LAT1, LAT2 and TAUT expression in placental trophoblasts. PPARγ modulation of LAT1 and TAUT is dependent on SP-1 signaling. The protein levels of PPARγ, SP-1, LAT1 and LAT2 are significantly downregulated in SGA placentas and correlate positively to birth weight in human placentas, indicating that PPARγ, SP-1, LAT1 and LAT2 in placentas are involved in control of fetal growth. PPARγ may be the therapeutic target for IUGR.

## Methods

### Ethics statement

The present study was approved by the specialty committee on ethics of biomedicine research, Second Military Medical University as well as ethics committee of Maternity and Child Health Hospital of Pudong New District. Written informed consent was obtained from all patients. Informed consent was obtained from all patients. Tissue collection was performed in accordance with the guidelines and regulations of the above committees. Data were analyzed anonymously.

### Tissue acquisition

A total of 48 placental specimens (16 from SGA, 14 from LGA and 17 from AGA infants) were collected at term delivery in Maternity and Child Health Hospital of Pudong New District, Shanghai, China during 2012–2013. Enrolled patients with SGA or LGA were fulfilled the following inclusion criteria: i) singleton pregnancy; ii) estimated fetal weight (EFW) ≤ or ≥ the 10th centile at the routine third trimester ultrasound^30^,^31^,^32^,^33^,^34^ weeks’ gestation), after adjustment for gestational age (GA) at delivery and gender according to local standards, and iii) normal UA Doppler at the time of diagnosis of SGA or LGA. The following exclusion criteria were considered: i) congenital or chromosomal abnormalities, ii) GA at delivery less than 34 weeks, iii) development of abnormal UA Doppler during follow-up, iv) assisted reproduction, concurrent disease such as hypertension and diabetes. Characteristics of subjects were shown in Table 1.

### Placental cell culture

Cultures of primary placental trophoblast cells were performed using the Kliman’s method^22^,^39^. Briefly, placental tissues obtained from healthy pregnancies at term were minced and dispersed with 0.25% trypsin (Invitrogen, Carlsbad, CA) and 0.1% deoxyribonuclease I (Sigma-Aldrich, St. Louis, MO). The purified cytotrophoblasts were separated by Percoll gradient centrifugation. The cells were then diluted with DMEM containing 10% fetal bovine serum (FBS), and then plated into 12-well plates at a density of 1.2 × 10^6^/well and cultured at 37 °C in 5%CO_2_-95% air.

After 48 h of plating, culture medium was replaced by FBS-free DMEM containing rosiglitazone (Sigma-Aldrich), GW9662 (Sigma-Aldrich), GW1929 (Sigma-Aldrich) or 15d-PGJ_2_ (Sigma-Aldrich) at the indicated concentrations. The above reagents were dissolved in DMSO and then diluted by DMEM. The vehicle control was maintained by culture media containing same volume of DMSO (typically 0.01%). Each treatment was performed in triplicate for each preparation of cells. The cell viability was assessed by MTT assay, which indicated that all the treatments had no impact on the cell viability (data not shown).

### Quantitative real-time RT-PCR

Total RNA was extracted from cells by using TRIzol reagent (Invitrogen) according to the manufacturer’s guide-lines. Two micrograms of RNA was reverse transcribed using superscript reverse transcriptase (Invitrogen) and stored at −20 °C. Quantitative real-time PCR was performed in duplicates using Mini Opticon Detector (Bio-rad). The primers were listed in supplemental Table 1. Reaction solution consisted of 40 ng diluted cDNA product, 0.1–0.3 μM of each paired primer, and 2 × PCR Master Mix (TaKaRa, Otsu, Japan). SYBR green (Hoffmann-La Roche Ltd, Basel, Switzerland) was used as detection dye. The conditions were optimized according to preliminary experiments to achieve linear relationships between initial RNA concentration and PCR product. The annealing temperature was set at 58 °C, and amplification cycles were set at 40 cycles. The temperature range to detect the melting temperature of the PCR product was set from 60–95 °C. The specificity of the primers was verified by both melting curve analysis and sequencing of the real-time RT-PCR products. Amplification of the housekeeping gene, β-actin and GAPDH were determined for sample loading and normalization. To determine the relative quantitation of gene expression for both target and housekeeping genes, the comparative Ct (threshold cycle) method with arithmetic formulae (2^−△△Ct^) was used^40^. Since very similar data were obtained by using either β-actin or GAPDH as an internal control, β-actin was used for calculation of ΔCt in presentation of the results.

### Western Blot Analysis

Placental tissues were homogenized in ice-cold RIPA lysis buffer containing phosphatase inhibitor cocktail (Roche, Indianapolis, IN), and the primary placental cytotrophoblast cells were scraped off the plate in the presence of the above buffer. 80 g proteins were heated at 99 °C for 5 min and then separated by SDS-PAGE (10%) and subsequently transferred to nitrocellulose membranes by electroblotting. Membranes were then blocked in 5% skimmed milk diluted in TBS-T (20 mMTris Base, pH7.6, 137 mM NaCl, 0.15% Tween-20) for 1 h at room temperature or overnight at 4 °C. Then membranes were incubated with antibodies recognizing LAT1 (Epitomics, Burlingame, CA), LAT2 (Santa Cruz Biotechnology, Inc. Santa Cruz, CA), TUAT (Millipore, Billerica, MA), SP-1(Epitomics), RXRα (Santa Cruz), PPARγ (Santa Cruz) at a dilution 1: 500–1000 overnight at 4 °C. Blots were washed three times for 10 min with TBS-T and probed with a HRP-conjugated secondary antibody at room temperature for 60 min. The intensities of light-emitting bands were detected and quantified using Sygene Bio Image system (Synoptics Ltd, London, UK). Total β-actin protein levels were detected for sample loading correction and normalization. In determination of the expression level of above protein, we had a bulk preparation of normal placental protein which was set as a control.

### RNA interferences

The sequence-specific small interfering RNA (siRNA) targeting human PPARγ (sense: 5′-CUGGCCUCCUUGAUGAAUATT-3′; antisense: 5′-UAUUCAUCAAGGAGGCCAGTT-3′), RXRα(sense: 5′-AGGACUGCCUGA-UUGACAATT-3′; antisense: 5′-UUGUCAAUCAGGCAGUCCUTG-3′) and SP-1 (sense: 5′-GGUCAUUUCUUUGCUUAUGTT-3′; antisense: 5′-CAUAAGCAA-AGAAAUGACCTT-3′) were designed and synthesized by Gene Pharma Corp (Shanghai, China). The negative control siRNA fragment is as followings: sense: 5′-UUCUCCGAACGUGUCACGUTT-3′; antisense: 5′-ACGUGACACGUUCGG- AGAATT-3′. The cultured primary placental trophoblast cells were transfected with the above fragments using Lipofectamine TM 2000 as described previously^22^.

### Assessment of *de novo* protein synthesis involvement

Cells were treated with the translational inhibitor cycloheximide (CHX) (Sigma-Aldrich) at 10^−5^ M for 1 h then following the addition of rosiglitazone (10^−6^ M). After 24 h treatment, the cells were harvested for RNA isolation and quantitative RT-PCR analysis.

### Determination of LAT2 promoter activity

The pGL3-luciferase reporter plasmid carrying LAT2 promoter (−5000 ~ +450 upstream to the transcription start site) was provided by Generay Biology Corp (Shanghai, China). LAT2 reporter plasmid and pRL-TK-Renilla-luciferase plasmid (Promega, Madison, WI) were transfected into trophoblasts using LipofectamineTM2000 at a dose of 0.5 ug/well and 50 ng/well, respectively. After 12 h, cells were treated with rosiglitazone (10^−6^ M) for 24 h. Luciferase assays were performed using dual luciferase assay kit (Promega). Relative luciferase activity is presented as firefly luciferase values normalized to renilla luciferase activity.

### Chromatin immunoprecipitation (ChIP)

ChIP was carried out using EZ Chromatin Immunoprecipitation Kit (Millipore) following the manufacturer’s protocol. Briefly, trophoblast cells (10^6^) were treated with 1% formaldehyde for 10 min at 37 °C to cross-link protein to DNA. The cross-linked cells were washed twice with PBS and once with IP buffer. Sonication is performed with eight 10-s pulses at 30% amplitude to shear the chromatin to a manageable size (about 200–1000 bp). After centrifuging, the supernatant was then immunoprecipitated with anti-SP-1 antibody or control immunoglobulin G. Immune complexes were precipitated by using salmon sperm DNA protein A-agarose and washed with IP buffer. Immunoprecipitated genomic DNA was eluted and used for real-time PCR with primers designed to amplify proximal promoter regions (supplementary Table 2).

### Statistical analysis

For illustration purpose, the data were normalized to the control and expressed as mean % of control ± SEM. All data were tested for homogeneity of variance by Bartlett’s test before comparisons. Individual comparisons were made by one-way ANOVA followed by LSD-t test. Pearson’s correlation was utilized to examine the correlation among PPARs, SP-1, amino acid transporters and birth weight. *P* ≤ 0.05 was considered to be significant.

## Additional Information

**How to cite this article**: Chen, Z. *et al.* PPARγ stimulates expression of L-type amino acid and taurine transporters in human placentas: the evidence of PPARγ regulating fetal growth. *Sci. Rep.* **5**, 12650; doi: 10.1038/srep12650 (2015).

## Supplementary Material

Supplementary Information

## Figures and Tables

**Table 1:** Clinical characteristics of the pregnant women enrolled in this study.

	**SGA(n = 16)**	**AGA(n = 17)**	**LGA(n = 18)**	**p**	**p SGAvs AGA**	**p SGAvs LGA**
Maternal age (y)	26 ± 1.05	26.8 ± 0.95	26 ± 1	ns	ns	ns
Maternal weight (kg)	64 ± 1.75	72 ± 2.77	72.7 ± 2.49	0.012	0.009	ns
BMI (kg/m^2^)	25.04 ± 1.62	27.75 ± 0.85	27.89 ± 0.98	0.029	0.021	ns
Primiparous (%)	94%	76.5%	93%			
Gestational age (wk)	37.67 ± 0.33	39.6 ± 0.19	39.7 ± 0.3	<0.001	<0.001	ns
Infant birth weight (g)	2191.2 ± 91	3355 ± 52.3	3819 ± 67.5	<0.001	<0.001	<0.001
Placental weight (g)	475 ± 18	651 ± 22	871 ± 24	<0.001	<0.001	<0.001
Infant birth weight/ Placental weight	4.62 ± 0.31	4.82 ± 0.42	4.49 ± 0.29	ns	ns	ns
