# Supplementary material for: PPARγ stimulates expression of L-type amino acid and taurine transporters in human placentas: the evidence of PPARγ regulating fetal growth
Source: Sci Rep. 2015 Jul 31;5:12650. doi: 10.1038/srep12650 (PMC4521151; doi:10.1038/srep12650)
Supplement: Supplementary Information [file srep12650-s1.doc]

**PPARγ stimulates expression of L-type amino acid and taurine transporters in human placentas: the evidence of PPARγ regulating fetal growth**

Zhaoguang Chen^1^, Ping He^1^, Xiaoying Ding^2^, Ying Huang^2^, Hang Gu^3^,Xin Ni^1*^

^1^Department of Physiology, Second Military Medical University, Shanghai, China; ^2^Maternity and Child Health Hospital of Pudong New District, Shanghai, China, ^3^Department of Obstetrics and Gynecology, Changhai Hospital, Shanghai, China

**Supplement materials**

Supplement Tab 1: The sequences of primers used in Realtime-PCR.

| Gene | Primer set (5’～3’) | | | Product size (bp) | | |
| --- | --- | --- | --- | --- | --- | --- |
| name | Sense | | Antisense |
| β-actin | TGTGTTGGCGTACAGGTCTTTG | | GGGAAATCGTGCGTGACATTA AG | | 275 | |
| SNAT 1 | CCAGTGGCCTAGCTGGTACCAC | | TCCCCAGCGAAAGTTGACTCAGAC | | | 204 |
| SNAT 2 | | ATCTGGCTGTAAGGTTTGCCCT | AAATGGACCAACGGTTTCACCC | | 198 | |
| SNAT 4 | | GTGAACTTAAAGATCGGTCCC | TATACACTTTGCTGTAGGCAT | | 168 | |
| LAT1 | | TTCATCGCAGTACATCGTGG | CCCAGGTGATAGTTCCCGAA | | 537 | |
| LAT2 | | AGCCCTGAAGAAAGAGATCG | TGCATATCTGTACAATCCCC | | 530 | |
| TAUT | | CTTTGTGTCTGGCTTCGCAATAT | GGGGTAGGCAATGAAGGCTAG | | 115 | |

Supplement Tab 2: The sequences of primers used in ChIP assay.

| sequence | Primer set (5’～3’) | | | Product size (bp) |
| --- | --- | --- | --- | --- |
| LAT1 | | Sense | Antisense |
| Primer 1 | | CTGGCTCACCCTGTCATGTCC | TCTGCCCTATTCTTCCCGCTGT | 374 |
| Primer 2 | | TATCTGTGTGACCTCCGCACT | GTCAGAATCCCTGTCGTGCT | 213 |
| Primer 3 | | GCTTCCAAGGACTCGTTTGCAC | AATACTGCCCCGAGATTGACC | 138 |
| Primer 4 | | CGCCTCTAACTGAAATTACCTCT | CCCCACCCCCAAATCTACCTG | 133 |
| Primer 5 | | CCCCAAACTCCATCCTTG | TCATCCCAGCCCGTCCTC | 146 |
| Primer 6 | | CACGCGGGCCTGGGAATAGC | GCATCTTCTCCCGCGCCTCTT | 337 |
| TAUT | |  |  |  |
| Primer 1 | | TTCACTGCTCGTGGCTTAG | TCTCCCACATCCCTCTGC | 207 |
| Primer 2 | | CAGTGGTCCTCGTCTGTCTT | AGGAGCATCCCGAAGTGA | 117 |
| Primer 3 | | GGTTCTCACAGCGTTTCCCT | TGCCAAGCCAGTGTCAGCTA | 149 |
| Primer 4 | | TCTCCCAGAACGTGGACGAT | TCACAGCAGCCTCCAAACTCC | 145 |
| Primer 5 | | TCTTTGTGTGTCCGAGCCCAT | TCTTCCTGACCTCGCGTGTCT | 218 |


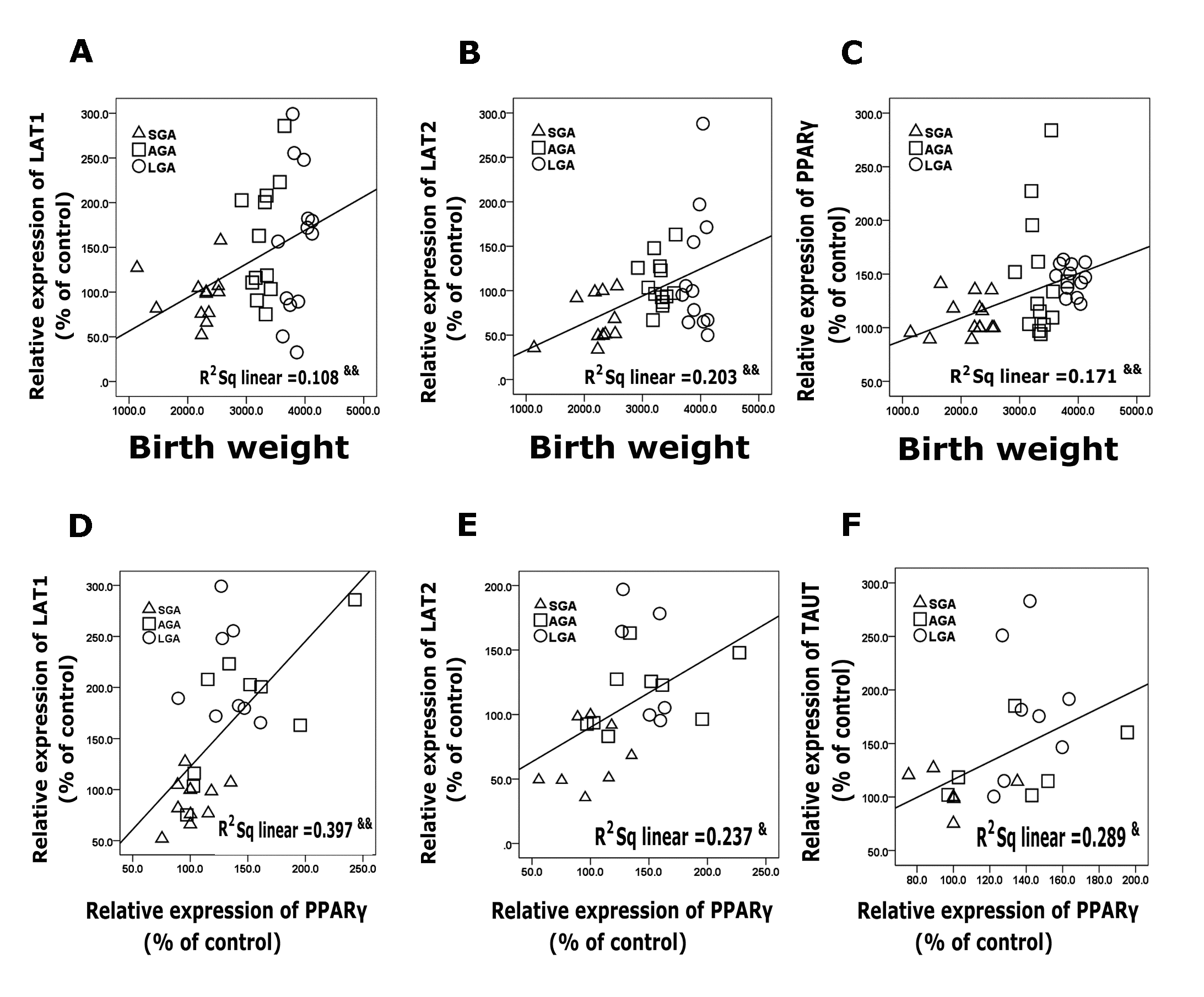


**Supplement Fig.1.**The relations between PPARγ and three amino acid transporters in placentas and their correlations with birth weight. A-C, there is a positive correlation between LAT1(A), LAT2(B) and PPARγ (C) protein expression and birth weight in human placenta. D-F, there is a positive correlation between LAT1(D), LAT2(E) and TAUT(F) protein expression and PPARγ protein expression in human placentas.&*P*<0.05. &&*P*<0.01.


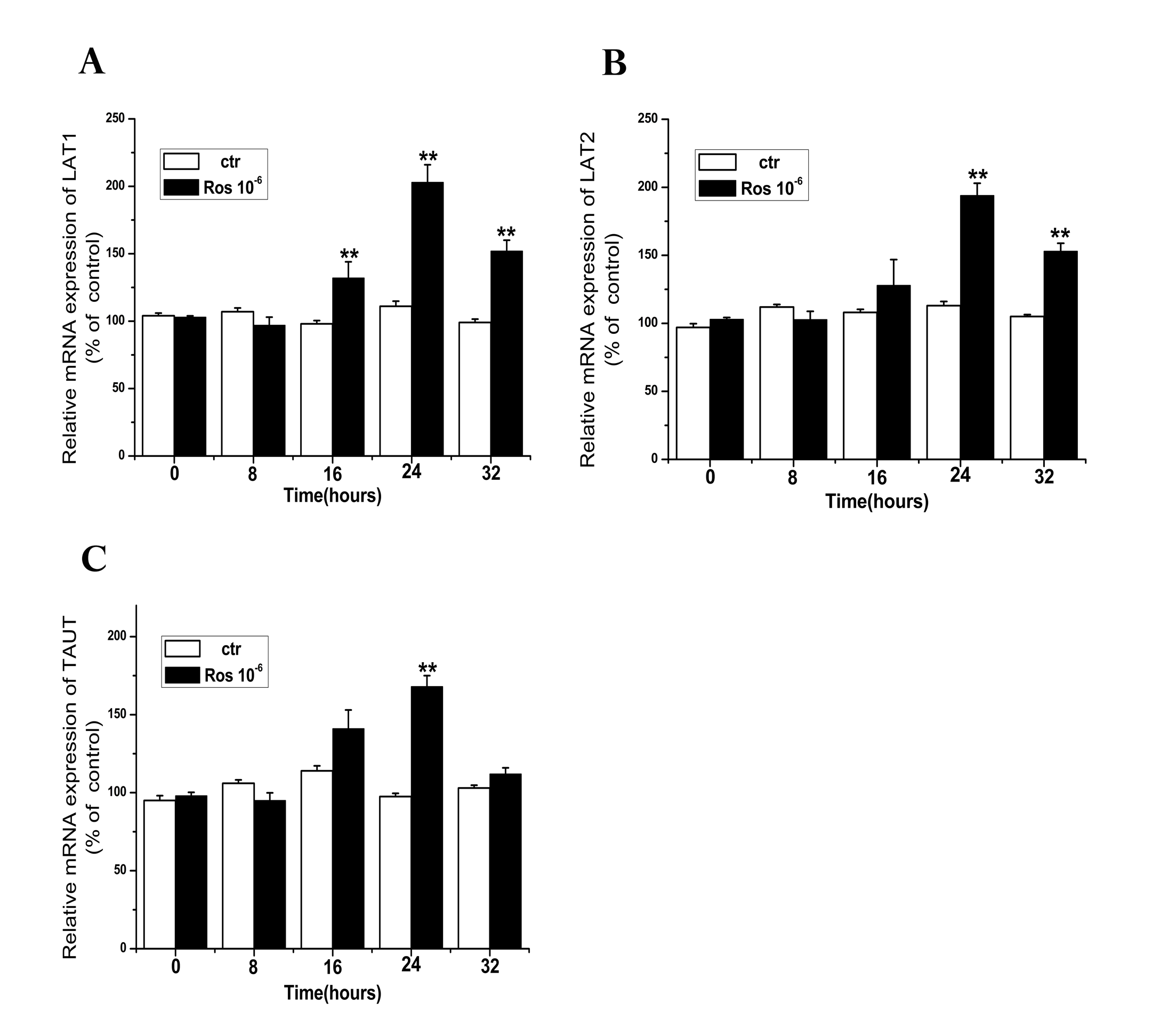


**Supplement Fig.2** The time course study of effects of rosiglitazone on LAT1, LAT2 and TAUT mRNA expression in cultured placental trophoblast cells. Cells were treated with rosiglitazone (10^-7^M) for 8-32h. Data are presented as mean percent control ± SEM of four cultures (n =4) performed in triplicate.Ros, rosiglitazone., ** *P*< 0.01 vs control





**Supplement Fig.3.**Effects of PPARγ agonist rosiglitazone on the expression of SNAT1、SNAT2 and SNAT 4 in human trophoblast cells. Cells were treated with rosiglitazone (10^-12^-10^-6^M) for 24h in the presence or absence of PPARγ antagonist GW9662(10^-6^M) for 24h.Representative protein bands are presented on the top of corresponding histogram.Data are presented as mean percent control ± SEM of five cultures (n = 5) performed in triplicate. Ros, rosiglitazone.


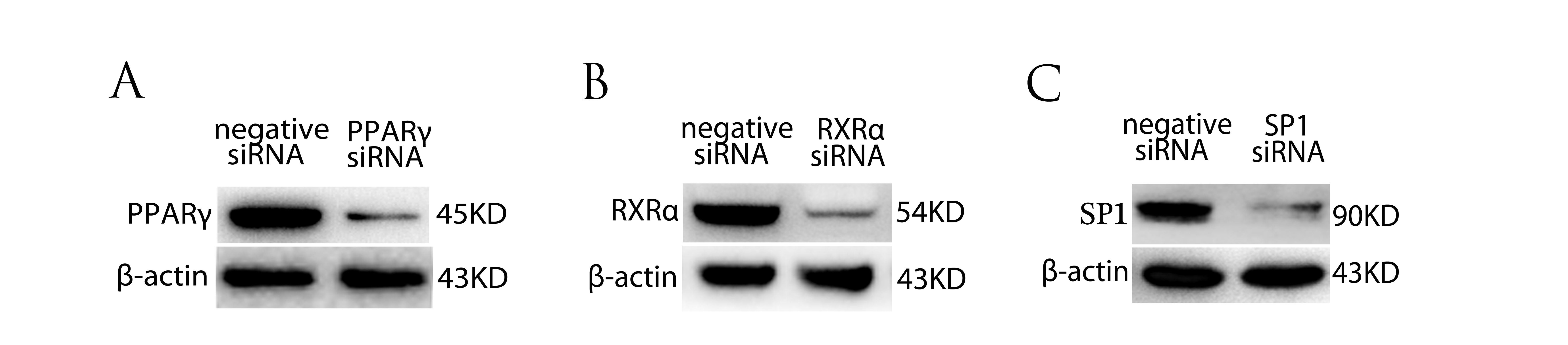


**Supplement Fig.4.**Representative bands for protein expression of PPARγ, RXRα and SP-1 in cells transfected with PPARγ, RXRα or SP-1 siRNA. A, cells were transfected with scramble siRNA or PPARγsiRNA. B, cells were transfected with scramble siRNA or RXRα siRNA. C, cells were transfected with scramble siRNA or SP-1 siRNA.
